# A mobile device-based imaging spectrometer for environmental monitoring by attaching a lightweight small module to a commercial digital camera

**DOI:** 10.1038/s41598-017-15848-x

**Published:** 2017-11-15

**Authors:** Fuhong Cai, Wen Lu, Wuxiong Shi, Sailing He

**Affiliations:** 1 Department of Electrical Engineering, Mechanical and Electrical Engineering College, Hainan University, Haikou, 570228 China; 2 Department of Biochemistry and molecular biology, Hainan Medical University, Haikou, 571001 China; 3 State Key Laboratory of Modern Optical Instrumentations, Centre for Optical and Electromagnetic Research, Zhejiang University, Hangzhou, Zhejiang, 310058 China

## Abstract

Spatially-explicit data are essential for remote sensing of ecological phenomena. Lately, recent innovations in mobile device platforms have led to an upsurge in on-site rapid detection. For instance, CMOS chips in smart phones and digital cameras serve as excellent sensors for scientific research. In this paper, a mobile device-based imaging spectrometer module (weighing about 99 g) is developed and equipped on a Single Lens Reflex camera. Utilizing this lightweight module, as well as commonly used photographic equipment, we demonstrate its utility through a series of on-site multispectral imaging, including ocean (or lake) water-color sensing and plant reflectance measurement. Based on the experiments we obtain 3D spectral image cubes, which can be further analyzed for environmental monitoring. Moreover, our system can be applied to many kinds of cameras, e.g., aerial camera and underwater camera. Therefore, any camera can be upgraded to an imaging spectrometer with the help of our miniaturized module. We believe it has the potential to become a versatile tool for on-site investigation into many applications.

## Introduction

In environmental monitoring fields, acquiring spectral and imaging information is essential to relate structure to function^1–3^. Imaging spectrometers are appealing instruments that provide a spectrally-resolved image of an object^4,5^. This spectrally-resolved image, also called a multispectral/hyperspectral image, is a promising tool currently applied to numerous areas, including environmental monitoring^6–8^, disease diagnosis^9^, food quality control^10^, ripeness testing^11^, etc. The imaging spectrometer is composed of two primary components: the detection and diffractive optical imaging systems. Not long ago, the spectrometer was restricted to laboratory applications due to its bulky size. Recently, considerable attention has been devoted to mobile devices because of their increasingly-sophisticated features. As an emerging imaging modality, the CCD/CMOS sensor in a mobile device provides efficient photoelectric conversion. As a result, many research teams have developed mobile spectrometers^12–16^. For example, Wei *et al.* detected mercury contamination in water using a smart phone^12^, and Das *et al,* developed a wireless smartphone spectrometer for fruit testing^13^. However, the existing mobile device-based spectrometer is currently not conducive to be further extended for multispectral imaging because it can only obtain the spectrum of one point on the sample. Lack of image information limits its utilization in remote sensing.

**Figure 1:**
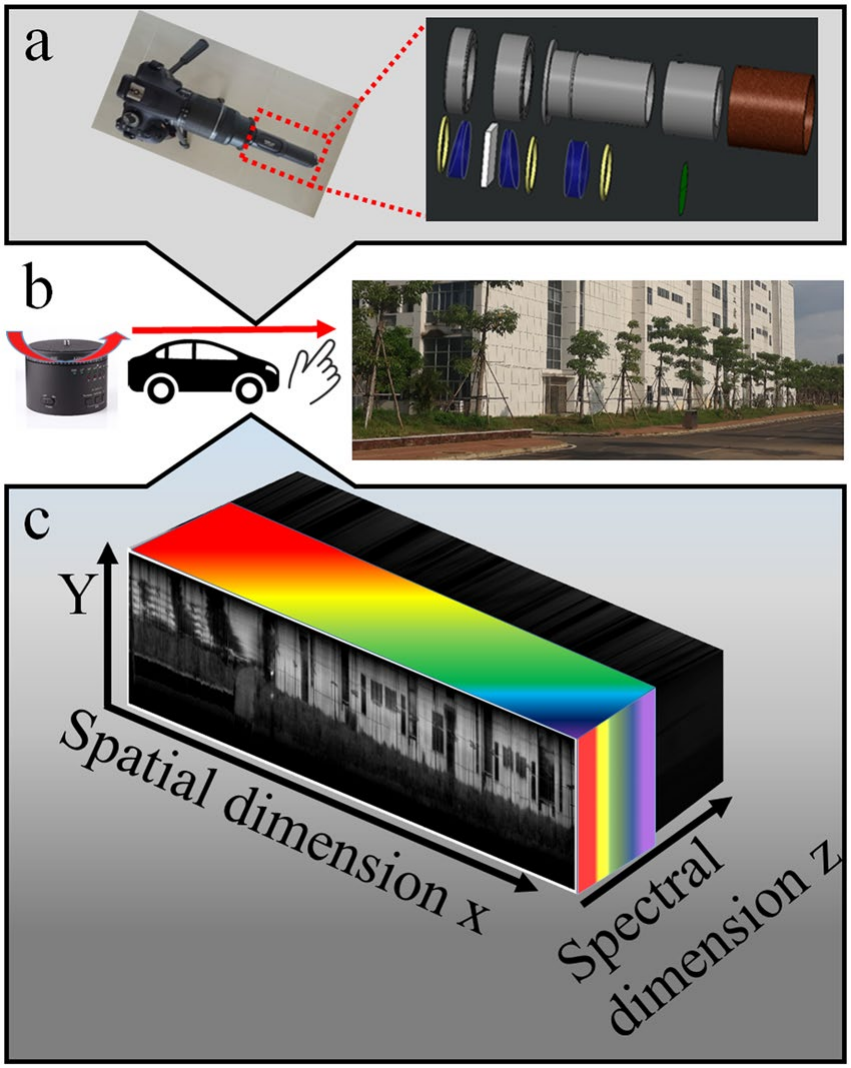
Illustration of the miniaturized imaging spectrometer equipped on a camera. (**a**) A photo of the prototype. (**b**) The operation modes for our imaging spectrometer, including rotation stage mode, vehicular mode and manual mode. At each mode, the imaging spectrometer yields a 3D spectral image cube. (**c**) A diagram of 3D spectral image cube, including two-dimensional spatial and one-dimensional spectral information.

In this study, we demonstrate a mobile-device based imaging spectrometer that is standalone and supported on a commercial digital camera. Because of its portability and ease of use, the digital camera plays an important role in scientific research^17^. Furthermore, a miniature unmanned rotorcraft equipped with digital cameras as a detection system has the ability to capture topographical images of a region of interest^18^. Incorporation of multispectral imaging into the commercial digital camera is worthy of development, as such development can allow the researcher to obtain the complete visible spectrum of a sample at any accessible place. As shown in Fig. 1a, we utilized a digital single lens reflex (SLR) camera as the sensor, and a miniaturized imaging spectrometer was connected to the SLR camera through a threaded adapter (Method and Supplementary Information give detailed information). This miniaturized imaging spectrometer is small and lightweight (~99 g). One of the reasons for selecting a SLR camera as detection module is that it offers a high sensitivity CMOS sensor for capturing spectral image. As a result, we can perform on-site multispectral imaging with the help of a motorized rotation stage or vehicle. Furthermore, this instrument can be hold by hand to perform push-broom multispectral imaging. During the imaging experiment, the imaging spectrometer is scanning along horizontal axis and the camera is worked at video mode. After wavelength calibration (see Method and Supplementary Information), each frame of video can be converted to many spectra, which are emitted from one vertical line region of a scanning object. Stitching all frames together, we can acquire a large amount of spectra from the scanning object surface. As shown in Fig. 1c, the scanning result can be converted to a 3D spectral image cube, involves two-dimensional spatial and one-dimensional spectral information. Integrating the 3D spectral image cube in spectral dimension, we can acquire an optical intensity image. Herein, by using this instrument in on-site experiments, we carried out water-color sensing and plant reflectance measurement, observed a satisfactory multispectral image and acquired reflectance spectra of several types of objects. Based on the experimentally results, we can monitor several important objects, such as chlorophyll in vegetation, phycocyanin and coloured dissolved organic matter (CDOM) in water, which previously must have been acquired by high-cost specialized instrument.

## Results

**Figure 2:**
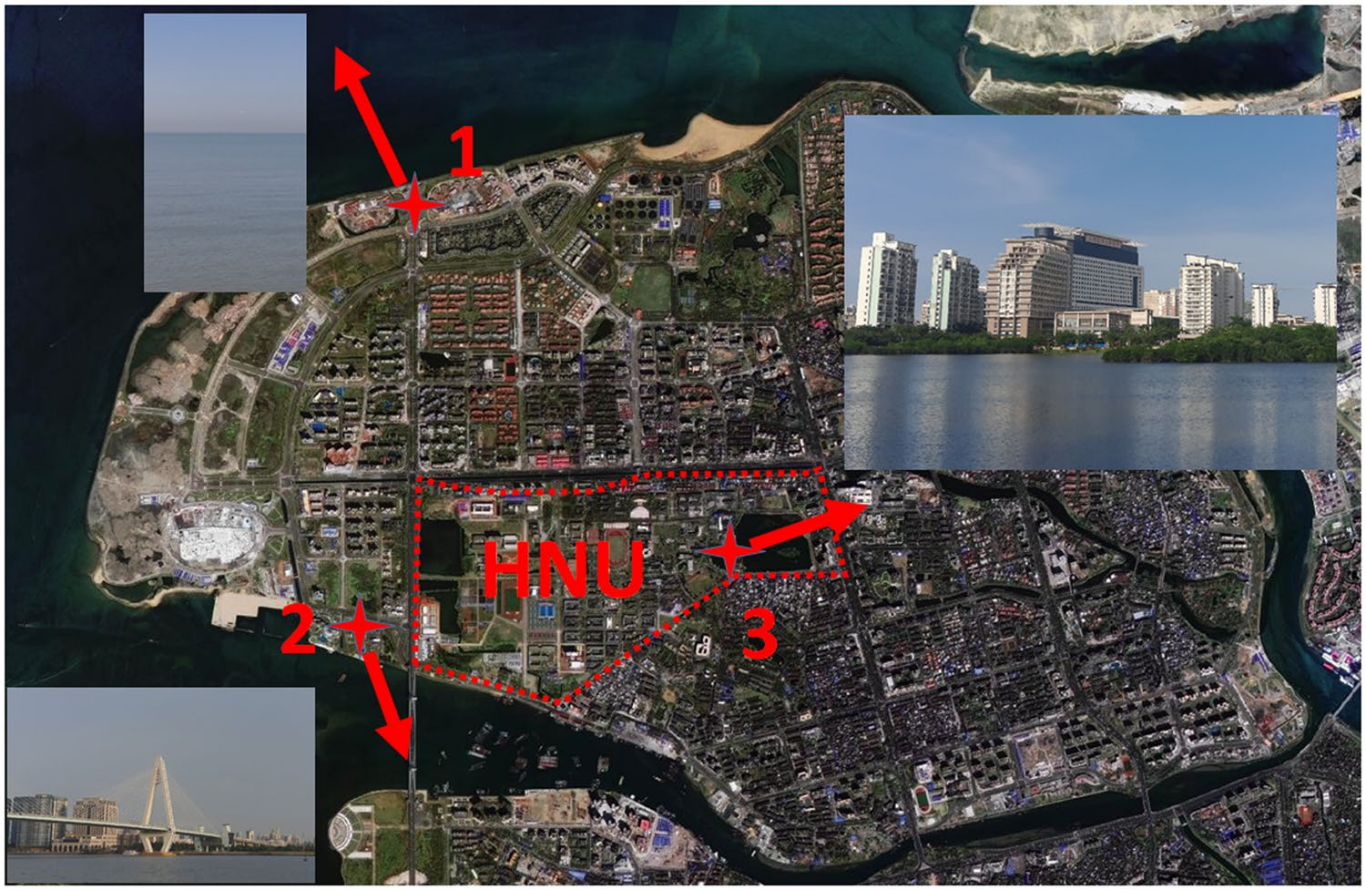
A satellite map of Haidian Island, Hainan province, China. The multispectral imaging experiments were performed at three locations: 1. Qiongzhou cape (N20.07°, E110.31°), 2. A sea crossing bridge (N20.05°, E110.31°), 3. Hainan University (HNU, N20.06°, E110.33°). Map was drawn by ArcGIS 9.3 (http://www.esri.com/arcgis/about-arcgis).

To assess the performance of our imaging spectrometer and demonstrate its ultra-mobile characteristic, we carried out a series of multispectral imaging at three locations within two hours. Figure 2 shows the location pins, which are a strait (location 1, known as Qiongzhou strait), a sea crossing bridge (location 2, known as Century Bridge) and Hainan University (location 3). The research objects involve ocean, lake, plants and buildings.

### Multispectral imaging for ocean

**Figure 3:**
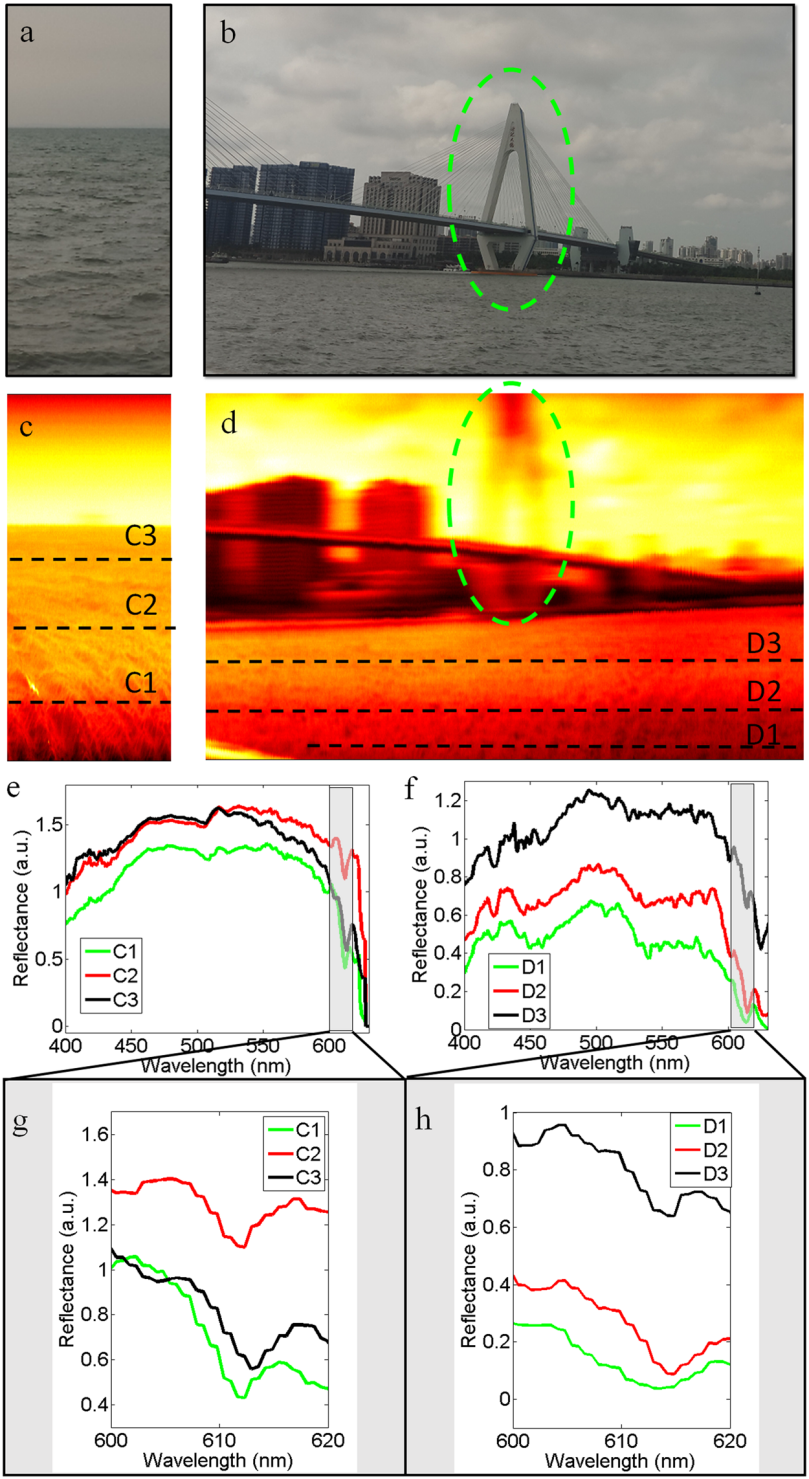
Multispectral imaging for ocean. (**a** and **b**) Show the photos of Qiongzhou strait and Century Bridge respectively. After multispectral imaging, 3D spectral image cubes can be obtained. (**c** and **d**) Illustrate the optical intensity images obtained from the 3D spectral image cubes. For each imaging object, we selected three line regions to study their reflectance of ocean surface. The reflectance spectra are shown in Figures (**e** and **f**). In order to study the concentration of phycocyanin, their reflectance spectra between 600 nm and 620 nm are shown in Figures (**g** and **h**), respectively.

Firstly, water-color sensing for ocean was performed at locations 1 and 2. The imaging spectrometer was placed on a motorized rotation stage (see Fig. S1c in Supplementary Information). As the stage was rotating around a horizontal axis, the camera worked at video mode. Each frame of video contained spectra emitted from one vertical line region of a scanning object. At locations 1 and 2, the scanning objects were Qiongzhou Strait and Century Bridge, which were shown in Fig. 3a,b, respectively. After multispectral imaging, two 3D spectral image cubes could be obtained. Based on the 3D spectral image cubes, we calculated the optical intensity images for Qiongzhou Strait and Century Bridge, which are shown in Fig. 3c,d, respectively. We can observe sea wave in Fig. 3c and buildings in Fig. 3d. These results illustrate the accuracy of the scanning results. Furthermore, the spectral information can pave the way for a deep investigation into the ocean. For each imaging object, we selected three horizontal line regions to study their reflectance of the ocean surface. Fig. 3(e,f) illustrates the reflectance spectra from the line regions of C1, C2 and C3 (D1, D2 and D3) in Fig. 3(c,d). These reflectance spectra can be utilized in water-color sensing. For example, the absorption between 610 nm and 620 nm is related to the existence of phycocyanin^19,20^. Hence, we can predict the concentration of phycocyanin based on the reflectance between 610 nm and 620 nm, which are shown in Fig. 3g,h. As shown in Fig. 3g, the reflectance spectra of C1 and C3 (near the coastline of the strait) suffers more absorption compared with reflectance spectra of C2 (in the center of strait). This is because human activity leads to the increase of algae near the coastline. The phycocyanin concentration at C1, C2 and C3 regions were estimated to be 150.9 μg/L, 85.5 μg/L and 145.14 μg/L, respectively (see Supplementary Information). Due to the same reason, a similar result can also be observed in Fig. 3h. With increase of distance from the coastline, the reflectance intensity between 610 nm and 620 nm increased from D1 to D3. The phycocyanin concentration at D1, D2 and D3 regions in Fig. 3d were estimated to be 190.26 μg/L, 174.10 μg/L and 102.66 μg/L, respectively (see Supplementary Information).

### Multispectral imaging for a lake

**Figure 4:**
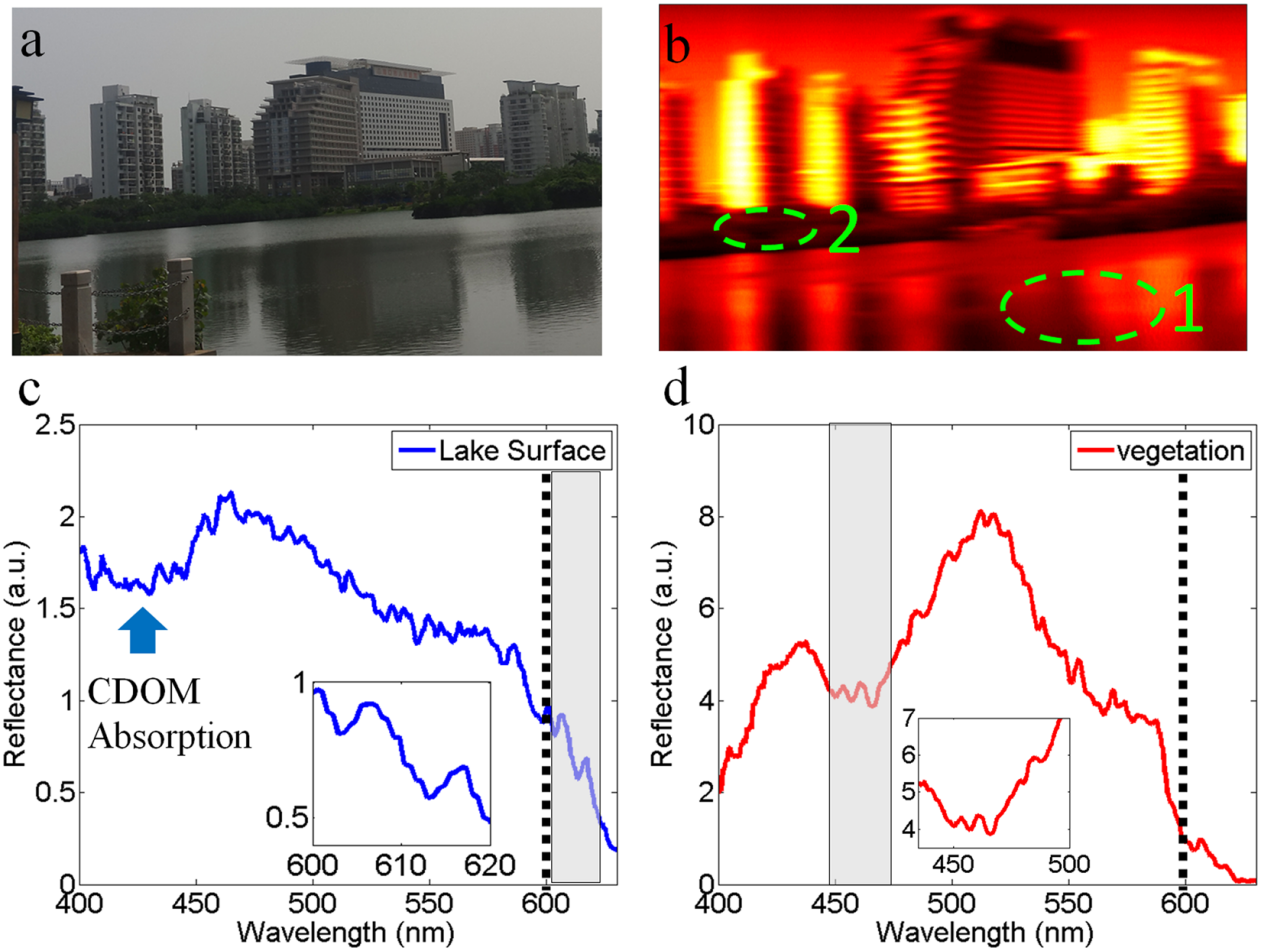
Multispectral imaging for a lake. (**a**) A photo of the scanning object. (**b**) The optical intensity image derived from the 3D spectral image cube. Dotted ellipse 1 and 2 denote lake surface and vegetation region respectively. (**c** and **d**) Show the reflectance from lake surface and vegetation region respectively. Inset of Figure (**c**) shows the reflectance of lake surface between 600 nm and 620 nm; Inset of Figure (**d**) shows the reflectance of vegetation between 440 nm and 500 nm.

Next, we could analyze the intrinsic characteristic of a lake at Hainan University through the multispectral imaging. The photo of the lake is shown in Fig. 4a. A similar scanning experiment based on our imaging spectrometer and motorized rotation stage was repeated to obtain a 3D spectral image cube. The corresponding optical intensity image is shown in Fig. 4b, from which we could identify the lake surface, vegetation and buildings. With the help of 3D spectral image data, the reflectance from the lake surface (dotted ellipse 1 region) and vegetation (dotted ellipse 2 region) could be easily extracted and shown in Fig. 4c,d respectively. Compared with the reflectance of the ocean surface, there was an obvious optical intensity attenuation in 400 nm–450 nm region. This is explained by the existence of coloured dissolved organic matter (CDOM) than ocean does^21^. Usually, a small lake contains more CDOM than ocean does. The absorption coefficient at 440 nm due to CDOM is 0.1586 m^−1^ (see Supplementary Information). In addition, due to the existence of phycocyanin in the lake, an absorption between 610 nm and 620 nm could be observed from the inset in Fig. 4c. The reflectance of vegetation is shown in Fig. 4d, and it’s known that chlorophyll in vegetation can absorb blue and red light. Hence, compared with the reflectance of the lake surface, the reflectance of vegetation exhibited a more sharply downward trend in > 600 nm range. The absorption of chlorophyll also led to optical intensity attenuation around 450 nm.

### Results of reflected spectra from flowers

**Figure 5:**
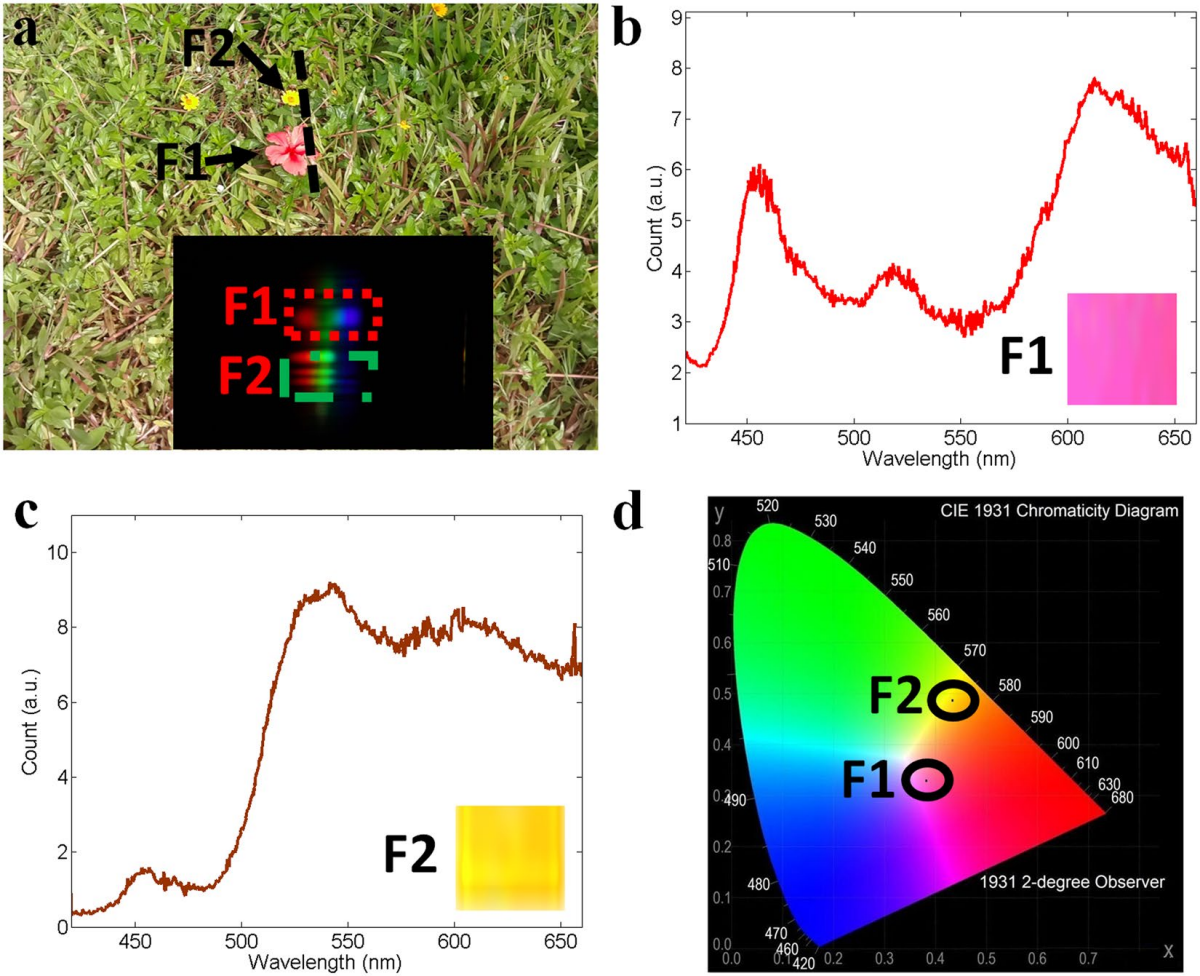
Results of reflected spectra from flowers. (**a**) A photo of the detected object; when the red and yellow flowers were in the camera field of view, a spectral photo was taken and shown as the inset. The ‘rainbows’ in boxes F1 and F2 represent the reflected spectra of the red and yellow flowers, respectively. After wavelength calibration, the real reflected spectra can be obtained and shown in Figures (**b** and **c**). According to these two reflected spectra, we can calculate the CIE coordinates, which are (0.3846, 0.3307) for the red flower and (0.4316, 0.4925) for the yellow flower. These two coordinates are indicated by the centers of ellipses shown in Figure (**d**). The estimated colors based on the CIE coordinates are shown as insets in Figures (**b** and **c**).

Since the imaging spectrometer module is extremely light (only about 99 g), we can hold our imaging spectrometer by hand to perform optical sensing for color measurement. As shown in Fig. 5a, there are two kinds of flowers in the grass by the lake. Adjusting the camera position until these two flowers fell in the camera field of view, then one photo was taken with an exposure time of 1/100 sec. The inset in Fig. 5a showed the captured photo, involving the spectral information of the red and yellow flowers. The reflected spectra arose from the red and yellow flowers were indicated by boxes F1 and F2. After wavelength calibration, accurate spectra were obtained and are shown in Fig. 5b,c, respectively. To verify the accuracy of the results, the reflected spectra were converted to CIE coordinates^22^, resulting in (0.3846, 0.3307) for the red flower and (0.4316, 0.4925) for the yellow flower. Figure 5d demonstrates the CIE chromaticity diagram, in which these calculated CIE coordinates were marked by the centers of ellipse F1 and F2. Based on the coordinates, we could find the estimated colors in CIE chromaticity diagram. The estimated colors are also shown as insets in Fig. 5b,c. These estimated colors are in conformity with the flowers’ colors. Additionally, a manual scanning spectral imaging for a river can be found in Supplementary Information S3.

### Multispectral imaging for street view

**Figure 6:**
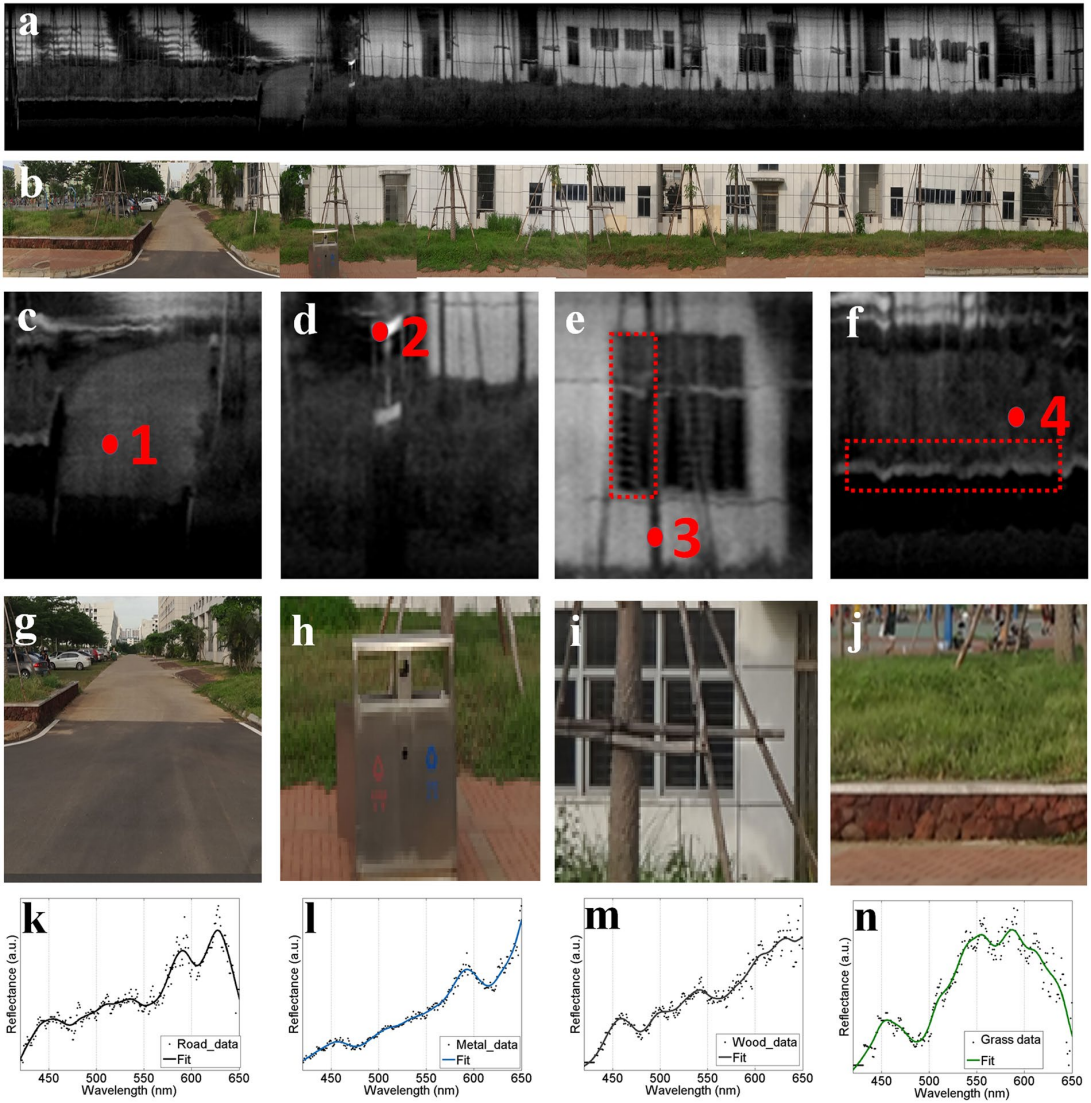
Results of multispectral imaging for street view via vehicular mode. (**a**) The optical intensity image derived from the 3D spectral image cube. (**b**) The panoramic photo for the street view. (**c**–**f**) Illustrate 4 partial features from the optical intensity image. (**g**–**j**) Show 4 color photos corresponding to Figures (**c**–**f**). In Figures (**c**–**f**), four features, including concrete in a road, a metal trash can, a sapling’s bark, and grass, were chosen to study their reflectance spectra, which are shown in Figures (**k**–**n**).

We can also perform multispectral imaging based on push-broom imaging method^23^ via vehicular mode. Herein, we carried out multispectral imaging experiment for street view^24^. Traditional street view provides panoramic views of the street. However, it lacks the spectral information for deep investigation, i.e. object recognition. In our work, the miniaturized imaging spectrometer was employed as a vehicle instrument. As the car was moving, the digital camera worked at video recording mode. The distance between the photographic subject and the camera was about 14 m. A 78-second video was taken and then converted to a 3D spectral image cube. Firstly, to illustrate the quality of the scanning results via vehicular mode, an optical intensity image was extracted from the 3D spectral image cube, as shown in Fig. 6a, and the panoramic photo of the street is shown in Fig. 6b. The optical intensity image closely matched the photo. For instance, we could identify a road (Fig. 6c,g), a metal trash can (Fig. 6d,h), saplings (Fig. 6e,i), and grass (Fig. 6f,j) in both Fig. 6a,b. In addition, as shown in Fig. 6e, the metal mesh behind a window can be distinguished, so the spatial resolution of the spectral image is better than the diameter of the metal mesh’s tube, which is 20 mm. It should be noted that, from the image of the road and metal trash can, there are image distortion and dither in the spectral image, which are due to a change in the vehicle’s speed and a bumpy road.

Furthermore, the spectral information enables an accurate study of the object in the street view. Figures 6k–n show the reflectance spectra for concrete in a road, a metal trash can, a sapling’s bark, and grass leaf. Different objects have different spectral characteristics, which are corroborated by previous studies^25–28^. In addition, these reflectance spectra can help perform an investigation into the characteristics of an interested substance. Taking the reflectance spectra of a grass leaf as an example, we observed one obvious drop off in intensity between 450 nm and 500 nm region, attributed to the absorption band of chlorophyll. Based on this kind of spectra, we can monitor the growth of plants along a greening road in future. Therefore, combined with our imaging spectrometer, a digital camera may serve as an easy-to-use and cost-effective piece of equipment for environmental monitoring that was not feasible previously.

## Discussion

In this work, we have presented the use of a digital camera as a mobile, portable sensor for environmental monitoring and sensing. Using an optomechanical tube that incorporates several small-size optical components and connecting them to the camera lens, this instrument can serve as a highly accurate imaging spectrometer for measuring the 3D spectral image cube from an object’s surface. Although the instrument is lightweight and small in size, it can achieve 12 nm spectral resolution and 20 mm spatial resolution at a distance of 14 m. Through investigation of these reflectance spectra contained in spectral image cube, we can acquire the unique characteristics for different objects. In this paper, the concentration of phycocyanin has been studied through the reflectance spectra between 600 nm and 620 nm. It’s shown that the reflectance from the center of the strait was higher than both sides of the strait. This is because the human activity led to the increase of algae near the coastline. Also, we observed an absorption around 400 nm–450 nm from reflectance of lake surface due to the existence of CDOM. Furthermore, based on the reflectance of plants, we can distinguish different kind of flowers, monitor the chlorophyll in vegetation.

The above results have shown that the combination of a commercial digital camera and our miniaturized imaging spectrometer module can be utilized as a mobile and portable optical sensing instrument. Based on this, our imaging spectrometer module may be assembled in a large number of equipment with cameras, e.g. unmanned aerial vehicles and provide ecologists a new tool for local-scale image capture. Additionally, our spectrometer module has the potential to be equipped on a smart phone for daily use, for instance meat property analyses^29^.

## Method

### The miniaturized imaging spectrometer module

All essential components of the module, such as the imaging lens, slit, doublet lens, and grating, have been assembled in an optical tube (length 105 mm and Ø31 mm). The entire system weighs about 99 g. A schematic illustration of the imaging spectrometer platform is shown in Fig. S1b in Supplementary Information. Briefly, the light passes through an imaging lens (f = 25 mm) and focuses on a slit (widths = 60 µm). A doublet lens (f = 50 mm) is used to collimate the light passing through the slit, then a grating (300 l/mm) is utilized to spread the spectrum over the SLR digital camera (with f = 55 mm camera lens). Herein, our imaging spectrometer is implemented on a SLR camera, which provides sensitivity to wavelengths in the visible range (~400 nm–650 nm). The exposure time and ISO were set as 1/100 sec and 1600, respectively, and the aperture for the camera lens is set as F5.6 during the scanning. When a digital photo is taken, a ‘rainbow’ spectral photo is obtained. One ‘rainbow’ spectral photo represents the spectra of one vertical line region of detected object and each horizontal array of the spectral photo contains the spectral data of one spatial point in the line region.

### Wavelength calibration

Each pixel in a horizontal array of the spectral photo represents one specific wavelength band. In order to obtain accurate spectral data, we need to correlate pixel index with a specific wavelength band. This process is performed by measuring a light source with known wavelengths. Then we can obtain several values of pixel index corresponding to the known wavelengths. Then calibration is carried out through reconstructing coefficients in a 3th order polynomial equation:

$${\rm{wavelength}}(\lambda )={{\rm{a}}}_{0}+{{\rm{a}}}_{1}x+{{\rm{a}}}_{2}{x}^{2}+{{\rm{a}}}_{3}{x}^{3}$$

where *x* is the pixel index value and a_0_, a_1_, a_2_, a_3_ are calibration coefficients whose values are 1916.78, −3.53, 0.00327 and −1.186 × 10^−6^. Supplementary Information gives a detailed information.

### The calculation of reflectance

Assuming the solar spectrum detected by our system is denoted as R_s(λ), the reflected spectrum from any object is denoted as R_f(λ). We then obtained the reflectance spectrum through R_f(λ)/R_s(λ).

### Data availability

All raw and processed imaging data generated in this work, including the representative images provided in the manuscript and Supplementary Information, are available from the authors upon request.

## Electronic supplementary material

Supplementary Information
