# Supplementary material for: A mobile device-based imaging spectrometer for environmental monitoring by attaching a lightweight small module to a commercial digital camera
Source: Sci Rep. 2017 Nov 15;7:15602. doi: 10.1038/s41598-017-15848-x (PMC5688121; doi:10.1038/s41598-017-15848-x)
Supplement: Supplementary file 1 — Supplementary Information [file 41598_2017_15848_MOESM1_ESM.doc]

**Supplementary Information**

A mobile device-based imaging spectrometer for environmental monitoring by attaching a lightweight small module to a commercial digital camera

Fuhong Cai^1^, Wen Lu^2*^, Wuxiong Shi^3^ and Sailing He,3[[1]](#footnote-2)*

^1^Department of Electrical Engineering, Mechanical and Electrical Engineering College, Hainan University, Haikou 570228, China

^2^Department of Biochemistry and molecular biology, Hainan Medical University, Haikou 571001, China

^3^State Key Laboratory of Modern Optical Instrumentations, Centre for Optical and Electromagnetic Research, Zhejiang University, Hangzhou, Zhejiang, 310058, China

**S1. The detail of a mobile device-based imaging spectrometer**

**
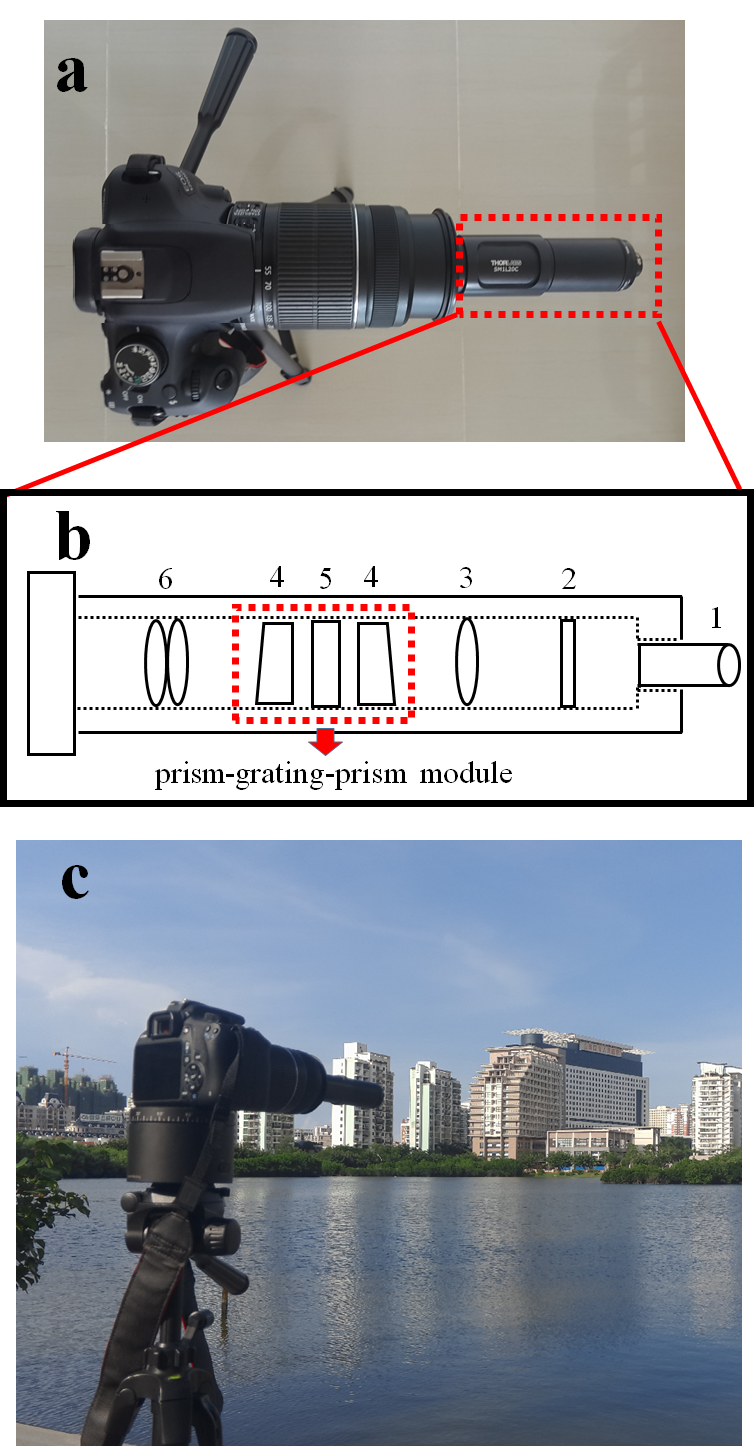
**

**Figure S1.** Illustration of the imaging spectrometer equipped on a camera. **a**. A photo of the prototype. **b.** Schematic diagram of the optical components, including: 1. Imaging lens; 2. Slit; 3. Doublet lens; 4. Wedge prisms; 5. Transmission grating; 6. The lens of the camera. **c.** A photo of the imaging spectrometer during experiment.

Detailed information about the imaging spectrometer system is described below. A HD CCTV camera lens was selected as the imaging lens. A custom-built slit ($20, 60 µm width, Ø25.4 mm) was placed at the image plane of the HD CCTV camera lens. These two components were housed in a tube (SM1L20, Thorlabs). Another slotted tube (SM1L20C, Thorlabs) which could be easily interfaced with SM1L20 was utilized to assemble a doublet lens (f = 50 mm) and a custom-built transmission grating ($80, 300 l/mm, Ø25.4 mm). This doublet lens was applied to collimate the light passing through the slit. The collimated light was then diffracted by the transmission grating. In order to correct the diffraction angle and allow the first order diffraction light to focus on the middle of the CMOS chip, two wedge prisms were used to construct a prism-grating-prism module (Herrala et al. 1994), as shown in Fig.S1b. The SM1L20C was connected to a camera lens (focal length: 55-250mm), which focused the first order diffraction light on the CMOS chip. The reason for using a SLR lens was that the focusing and zooming can be expediently performed to obtain a high-resolution spectral image.

Furthermore, the installation of the prism-grating-prism module should be addressed. We needed to rotate the wedge prisms so that the wedges of both prisms were aligned. In addition, the orientation of the grating grooves needed to be parallel to the slit. Hence the prisms and grating were housed in SM1L20C, which was a slotted tube and allowed for convenient rotation of the optical element.

Another feature of this system is the focusing of the CCTV camera lens, whose image plane coincided with the slit. Achieving this objective required a three-step process: firstly, a rough adjustment carried out to place the slit around the image plane of the CCTV camera lens according to its focal length. Secondly, we installed the rest of the system based on the above procedure. Thirdly, while the camera was aimed at a luminous object, we adjusted the position of the CCTV camera lens until a high-resolution image was shown in the display of camera.

The scanning video can be converted to a 3D spectral image cube, which involves two-dimensional spatial (X-Y axes) and one-dimensional spectral information (Z axis). Assuming the imaging spectrometer scans along Y axis, then each fame of a video can be defined as a rainbow spectral image, which represents a full spectrum from a line region (along X-axis) of the detected object (2D picture in X-Y space). This rainbow spectral image forms a slice image perpendicular to Y axis. With line-scanning along Y axis, a 3D spectral image cube can be obtained.

**S2. The wavelength calibration**


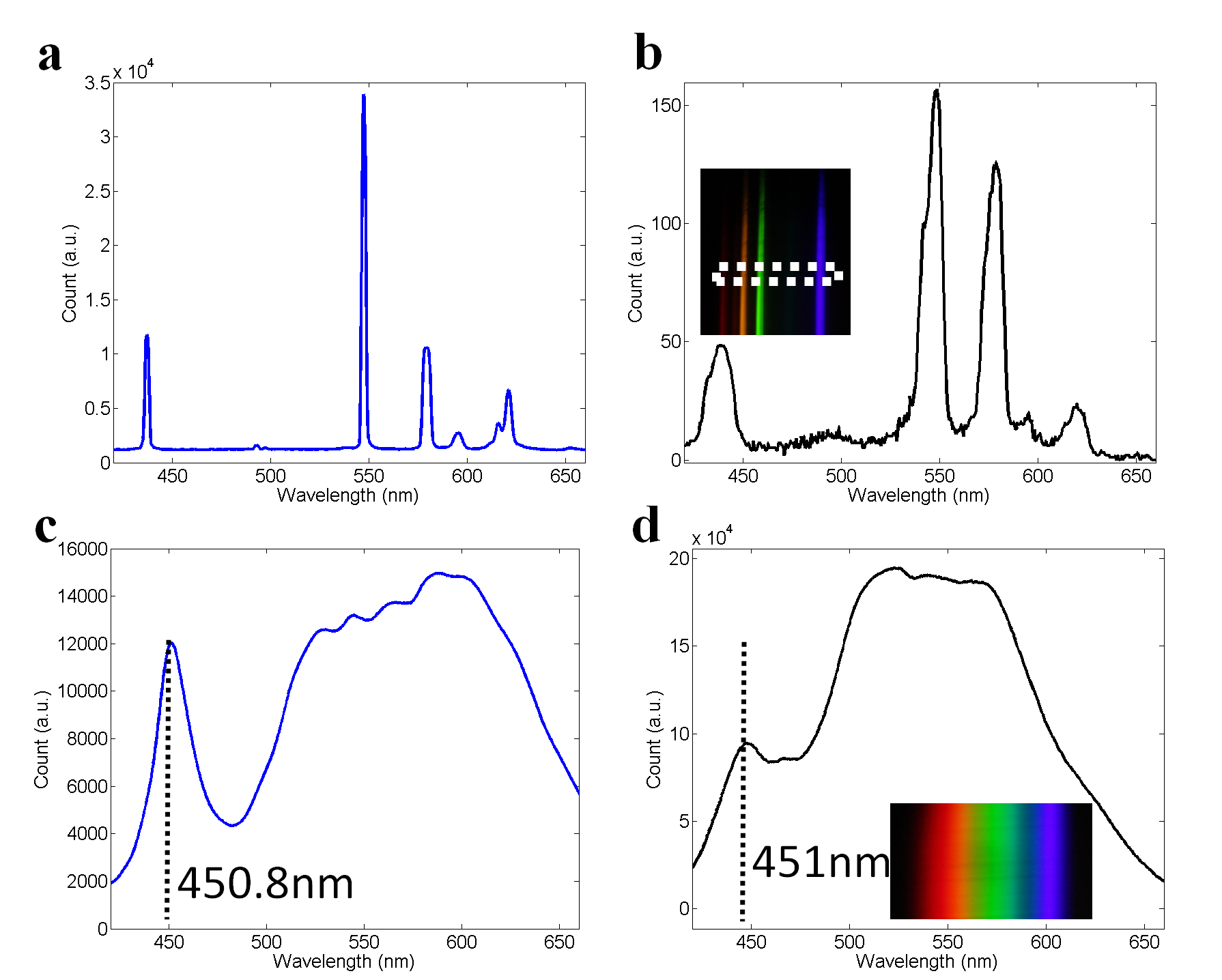


**Figure S2.** Optical spectra of a mercury lamp and white-light LED lamp. **a.** Accurate spectrum of the mercury lamp detected by a high spectral resolution commercial spectrometer. **b.** Spectrum of the mercury lamp detected by our system; the original spectral photo is shown as an inset. **c.** Accurate spectrum of the white-light LED detected by a commercial spectrometer. **d.** Spectrum of the white-light LED detected by our system; the original spectral photo is shown as an inset.

In order to obtain accurate spectral data, the system needed to be calibrated to correlate pixel index with a specific wavelength band. The wavelength calibration procedure is introduced below. Firstly, a mercury lamp, whose spectrum was measured by a high spectral resolution commercial spectrometer and is shown in Fig.S2a, was utilized as the standard light. The inset in Fig.S2b shows a photo derived from using our system to measure the light of the mercury lamp. This photo contains five vertical lines (red, lighter red, orange, green, and blue lines), which represent 620 nm, 596 nm, 578 nm, 548 nm, and 437 nm light from the mercury lamp (Sansonetti et al. 1996). The horizontal pixel index values of these five lines are 1392, 1458, 1503, 1595 and 1942. It should be noted that, since the vertical lines for 596 nm and 578 nm light can be separated, the spectral resolution of the system is better than 18 nm (596 nm – 578 nm = 18 nm). Combining the horizontal pixel index values and the wavelength values of these five vertical lines, the horizontal pixel index value can be translated to wavelength value according to a 3th order polynomial fitting (Wollman and Bohn 1993). The 3th order polynomial equation is shown as below:

$\text{wavelength}(\lambda)=\text{a}_{0}+\text{a}_{1}x+\text{a}_{2}x^{2}+\text{a}_{3}x^{3}$ (Eq.1)

where$x$is the horizontal pixel index value. In this manner, each pixel along the horizontal axis corresponds to a specific wavelength band. A subarea of the photo, indicated by the white box in the inset, was then chosen to plot a spectral curve, as shown in Fig.S2b. Herein we converted this color photo into a gray image. The gray value of each pixel was used as the optical intensity for the related wavelength band. In order to test the wavelength calibration procedure, our system was also applied to measure a white-light LED lamp. Fig.S2c showed an accurate spectrum of this LED. A ‘rainbow’ photo for this LED lamp is show as an inset in Fig.S2d. Based on the wavelength calibration procedure, we obtained the spectrum for the LED lamp, as shown in Fig.S2d. In Fig.S2c and S2d, we observe that both spectra show peak intensity values at wavelengths of 451 nm, indicating the reliability of the wavelength calibration procedure. It is worth noting that the full width at half maximum (FWHM) of the 548 nm band in Fig.S2a (detected by a commercial spectrometer) is about 2 nm. This spectral band in Fig.S2b (detected by our instrument) is broadened to 12 nm. Also note, from the above discussion, that the spectral resolution of the imaging spectrometer is better than 18 nm. Therefore, the spectral resolution of the system can be estimated to be 12 nm.

We form the image cube by using Matlab code. We utilized “VideoReader” function to load the video into the workspace of Matlab. In the workspace, the video was automatically converted to a serials of frames. As shown in Fig. S2b, the spectral image is smaller than the CMOS sensor. Then from each fame of a video we extracted this subarea, which is filled with the spectral signal, as a rainbow spectral image. All frames of the video were used to form a 3D spectral image cube.

**S3. Multispectral imaging for a river based on manual scanning**


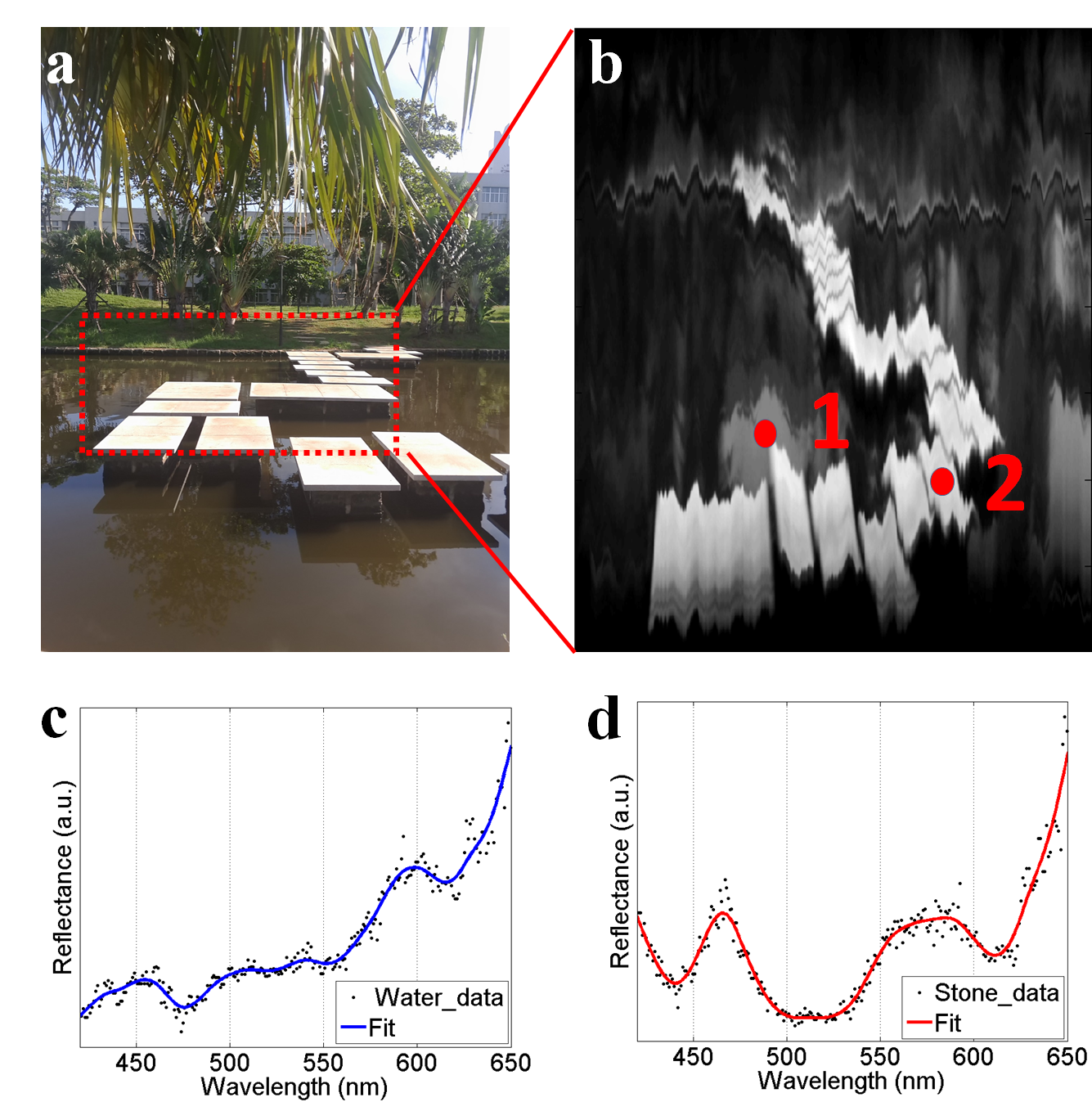


**Figure S3.** Results of manual scanning multispectral imaging for a river. **a.** A photo of the river; a manual scanning experiment was performed aimed at the red box and a 3D spectral image cube was obtained. **b.** The optical intensity image derived from the spectral image cube. Two spots, indicating water and stone, were chosen in Figure b, and their reflectance spectra are shown in Figure **c** and **d**, respectively.

As shown in Fig.S3a, we sought to perform a manual multispectral imaging experiment. Digital cameras are a commonly-used tool in environmental monitoring. Given our imaging spectrometer’s advantages in weight and size, it can be employed as a portable system. In addition, it is a standalone system and easy to connect to a digital camera. Therefore, our imaging spectrometer may work at manual mode. To testify our prediction, the imaging spectrometer system was held by hand to scan a river, as shown in Fig.S3a. During the scanning photograph, the camera worked at video recording mode, and a 13-second video was captured. This video could be translated to a spectral image cube, whose optical intensity image is shown in Fig. S3b. Owing to our amateur camera skills, the optical intensity image suffered from dithering. Nevertheless, we distinguish river bank and stone bridge in the image. Moreover, the reflectance spectra for the river water and stone bridge were obtained, as shown in Fig.S3c and S3d, respectively. For pure water, the reflectance spectrum exhibits a downward trend in visible light bands due to its absorption characteristics. In contrast, herein the river water shows a high reflectivity in the 550 nm to 650 nm band. This difference may be attributed to the fact that this river water is stagnant and muddy, as shown in Fig.S3a.

**S4. Reflectance for phycocyanin aqueous solution**


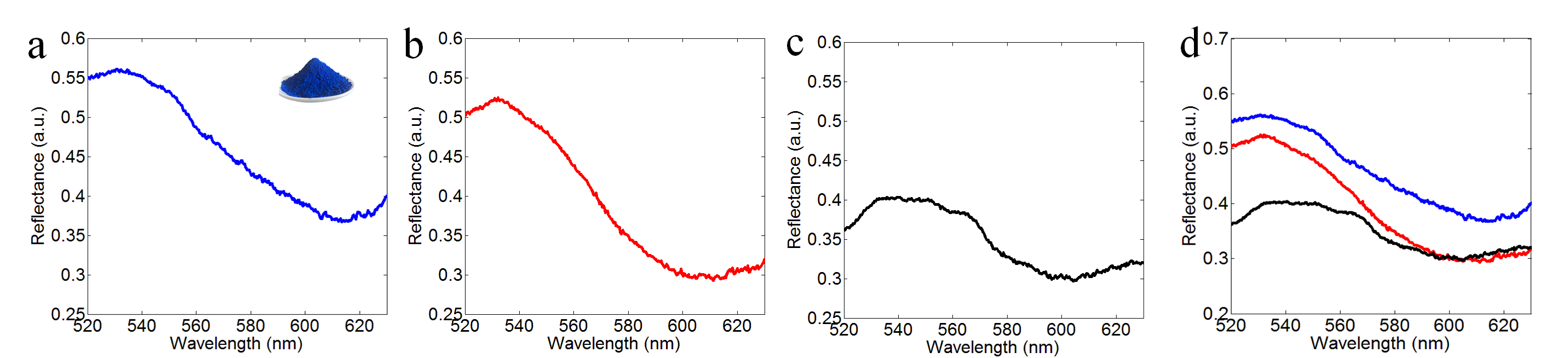


**Figure S4.** The reflectance for phycocyanin aqueous solution. (a) The reflectance spectrum detected at lab for 333 μg/L phycocyanin aqueous solution; (b) The reflectance spectrum detected at lab for 400 μg/L phycocyanin aqueous solution. (c) The reflectance spectrum detected at lab for 80 μg/L phycocyanin aqueous solution. (d) We merged the reflectance in (a) – (c) together for comparison.

In Ref.[20] (*Process Biochemistry* **41**, 2017-2023 (2006)), Soni et.al. measured the absorption spectra of phycocyanin at each stage of purification. The absorption peak varied from 600 nm to 620 nm. Therefore, the main absorption between 600 nm to 620 nm should derive from the phycocyanin. We measured in our laboratory the actual spectra from phycocyanin aqueous solutions of various concentrations under controlled conditions to support the data captured by our system. Firstly, 1000 μg phycocyanin powder was dispersed in 3 L water, then we measured the reflectance by our imaging spectrometer, and the result was shown in Fig.S4a. Then 1200 μg phycocyanin was dispersed in 3 L water, and the reflectance was shown in Fig.S4b. The phycocyanin concentration is related to the following formula (Ogashawara et. al. 2013):

$\text{A}\times\mathrm{R}(\lambda_{1})\text{/}\mathrm{R}(\lambda_{2})\text{+B}$ Eq.(2)

where λ_1_ is absorption peak wavelength around 612 nm, λ_2_ is selected as 535 nm in this wok. Based on the reflectance in Fig.S4a and S4b, the coefficient A and B are estimated to be -185.19 and 210.85. Next the phycocyanin aqueous solution was diluted to 80 μg/L, and the reflectance is shown in Fig.S4c. Based on Eq. (2) the phycocyanin concentration is estimated to be 69 μg/L, and the relative error is about 13.7%. We believe the error was due to the impurity in phycocyanin powder. It is also worth noting that impurity in the phycocyanin powder leads to blue shift in the absorption. Nevertheless, we can still utilize Eq. (2) to give a rough prediction of phycocyanin concentration based on the reflectance. Hence, the phycocyanin concentration at C1, C2 and C3 regions in Fig.3c were estimated to be 150.9 μg/L, 85.5 μg/L and 145.14 μg/L, respectively. Similarly, the phycocyanin concentration at D1, D2 and D3 regions in Fig.3d were estimated to be 190.26 μg/L, 174.10 μg/L and 102.66 μg/L, respectively.

The reflectance in c (80 μg/L) is relatively weaker than the reflectance in a (333 μg/L) and b (400 μg/L). This is due to relatively higher scattering caused by high concentration of phycocyanin. Scattering can change the propagation path of light in water, resulting in a small change in the shape of the absorption spectrum.

**S5. The estimation of absorption coefficient of coloured dissolved organic matter (CDOM)**

Due to the complexity of CDOM, it is hard to determine its concentration. The traditional method is to use the absorption coefficients of 355 nm, 375 nm or 440 nm to represent the concentration of CDOM. (Zhang et. al. 2007)

According to the appendix of Ref (Craig et. al. 2006), the absorption coefficient for CDOM is a(440 nm) = 0.1586 m^-1^, where a(λ) is the light absorption coefficient at wavelength λ. The absorption coefficient a(λ) can be estimated by:

$a(\lambda_{0})=0.0596+0.2[a(\lambda_{0})_{i}-0.01]$ (Eq.3)

where $a(\lambda_{0})_{i}\text{=}\exp(-0.62-1.97\rho+0.79\rho^{2})$,$\rho\text{=Ln}(\mathrm{rs}(\lambda_{0})/\mathrm{rs}(550))$,$\mathrm{rs}(\lambda)\text{=R}(\lambda)/(0.52+1.7\text{R}(\lambda))$.

Here$\text{R}(\lambda)$ represents the reflectance at wavelength

**References**

Herrala, E., Okkonen, J., Hyvarinen, T., Aikio, M., & Lammasniemi, J. (1994). Imaging spectrometer for process industry applications. In, *Proc. SPIE* (pp. 33-40)

Sansonetti, C.J., Salit, M.L., & Reader, J. (1996). Wavelengths of spectral lines in mercury pencil lamps. *Applied optics, 35*, 74-77.

Wollman, S., & Bohn, P. (1993). Evaluation of polynomial fitting functions for use with CCD arrays in Raman spectroscopy. *Applied spectroscopy, 47*, 125-126.

Ogashawara, I., Mishra, D. R., Mishra, S., Curtarelli, M. P., & Stech, J. L. (2013). A performance review of reflectance based algorithms for predicting phycocyanin concentrations in inland waters. *Remote Sensing, 5*, 4774-4798.

Zhang, Y., Zhang, B., Wang, X., Li, J., Feng, S., Zhao, et. al. (2007). A study of absorption characteristics of chromophoric dissolved organic matter and particles in Lake Taihu, China. *Hydrobiologia, 592,* 105-120.

Craig, S. E., Lohrenz, S. E., Lee, Z., Mahoney, K. L., Kirkpatrick, G. J., Schofield, O. M., & Steward, R. G. (2006). Use of hyperspectral remote sensing reflectance for detection and assessment of the harmful alga, Karenia brevis. *Applied Optics, 45,* 5414-5425.

1. ^^ Email for the corresponding author: wen_lu@yeah.net or sailing@kth.se [↑](#footnote-ref-2)
